# Exosomes derived from syncytia induced by SARS-2-S promote the proliferation and metastasis of hepatocellular carcinoma cells

**DOI:** 10.3389/fcimb.2024.1415356

**Published:** 2025-01-08

**Authors:** Huilong Li, Haotian Lin, Tinghui Fan, Linfei Huang, Li Zhou, Xiaoyu Tian, Ruzhou Zhao, Yanhong Zhang, Xiaopan Yang, Luming Wan, Hui Zhong, Nan Jiang, Congwen Wei, Wei Chen, Lihua Hou

**Affiliations:** ^1^ College of Basic Medical Sciences, School of Medicine, Zhejiang University, Hangzhou, China; ^2^ Department of Genetic engineering, Beijing Institute of Biotechnology, Beijing, China; ^3^ Department of Pharmacy, Medical Supplies Center of People’s Liberation Army (PLA) General Hospital, Beijing, China

**Keywords:** SARS-2-S, syncytia, exosomes, hepatocellular carcinoma, proliferation, metastasis

## Abstract

**Introduction:**

Coronavirus disease 2019 (COVID-19) is characterized by fever, fatigue, dry cough, dyspnea, mild pneumonia and acute lung injury (ALI), which can lead to acute respiratory distress syndrome (ARDS), and SARS-CoV-2 can accelerate tumor progression. However, the molecular mechanism for the increased mortality in cancer patients infected with COVID-19 is unclear.

**Methods:**

Colony formation and wound healing assays were performed on Huh-7 cells cocultured with syncytia. Exosomes were purified from the cell supernatant and verified by nanoparticle tracking analysis (NTA), Western blot (WB) analysis and scanning electron microscopy (SEM). Differentially expressed proteins in syncytia-derived exosomes (Syn-Exos) and their functions was analyzed by Proteomic sequencing. Syn-Exo-mediated promotion of hepatocellular carcinoma cells was measured by CCK-8 and Transwell migration assays. The mechanism by which Syn-Exos promote tumor growth was analyzed by Western blotting. A patient-derived xenotransplantation (PDX) mouse model was constructed to evaluate the pathological role of the SARS-CoV-2 spike protein (SARS-2-S). The number of syncytia in the tumor tissue sections was determined by immunofluorescence analysis.

**Results:**

Syncytium formation promoted the proliferation and migration of hepatocellular carcinoma cells. Proteomic sequencing revealed that proteins that regulate cell proliferation and metastasis in Syn-Exos were significantly upregulated. Syn-Exos promote the proliferation and migration of hepatocellular carcinoma cells. Animal experiments showed that a pseudotyped lentivirus bearing SARS-2-S (SARS-2-Spp) promoted tumor development in PDX mice. More syncytia were found in tumor tissue from SARS-2-Spp mice than from VSV-Gpp mice.

**Conclusions:**

Syn-Exos induced by SARS-2-S can promote the proliferation and metastasis of hepatocellular carcinoma cells.

## Introduction

Severe acute respiratory syndrome coronavirus 2 (SARS-CoV-2) broke out worldwide in 2019, resulting in unprecedented consequences. The mechanism by which SARS-CoV-2 infects the host involves the binding of the SARS-CoV-2 S (SARS-2-S) protein to the ACE2 receptor on the surface of the host cell. The virus fuses with the cell membrane and releases its genome into the cell (Benton et al., 2020). Coronavirus disease 2019 (COVID-19) is characterized by fever, fatigue, dry cough, dyspnea, mild pneumonia and acute lung injury (ALI) and can lead to acute respiratory distress syndrome (ARDS) (Sun et al., 2021b). Previous studies have shown that SARS-CoV-2 accelerates tumor progression by changing the metabolic pathway of tumor cells. Infection of human colonic epithelial cancer cell lines with SARS-CoV-2 reshaped critical cellular pathways, including translation, splicing, carbon metabolism and nucleic acid metabolism pathways (Kim et al., 2020). In addition, cancer patients usually have low immunity due to immune cell exhaustion and side effects of chemotherapy or radiotherapy (Sharma et al., 2024). The immune system of these patients is further damaged after initial infection with SARS-CoV-2. Compared with those in healthy individuals, the abundance of neutrophils and macrophages in COVID-19 patients was significantly increased, which could induce a strong inflammatory response (Xiong and Sun, 2023). Acute and chronic inflammation are risk factors for tumor progression, and the inflammatory microenvironment is a key factor in tumorigenesis (Zhao et al., 2021; Thorsson et al., 2018). In a study on SARS-CoV-2, the authors focused on a vulnerable group of cancer patients (Sun et al., 2021a). Recent studies have shown that cancer patients have a greater risk of severe COVID-19 and mortality than the general population (Leyfman et al., 2023; Fendler et al., 2022). At present, studies on the mechanism of COVID-19 in cancer patients are rare.

Syncytia refers to a multinuclear cell structure formed by the fusion of multiple cells. Syncytial formation usually involves the fusion of cell membranes, resulting in multiple nuclei within the same cytoplasm (Leroy et al., 2020). A previous report showed that SARS-CoV-2-infected cells form syncytia, and this formation is mediated by the binding of SARS-2-S and ACE2 (Buchrieser et al., 2020). However, exosomes derived from syncytia (Syn-Exos) have not been reported. Exosomes are vesicles that are 30-150 nm in size and contain complex components. They were first identified in sheep reticulocytes (Johnstone et al., 1987). Studies have shown that exosomes play a key role in the interactions between pathogens and hosts (Schorey et al., 2015). The pathogenesis of many viruses is closely associated with exosomes. For example, HIV activates latent viruses through exosomal Nef, thereby promoting apoptosis in CD4^+^ T cells and enhancing susceptibility to and transmission of the virus (Lenassi et al., 2010; Arenaccio et al., 2015). HCV is transmitted to neighboring cells through exosomes carrying viral genomes (Ramakrishnaiah et al., 2013). MiR-483-3p in serum exosomes is related to the pathogenesis of H5N1 influenza virus infection (Maemura et al., 2020). In recent years, in-depth studies have shown that exosomes participate in signal communication and have been at the forefront of cancer research in the past decade (Demory Beckler et al., 2013; He et al., 2021). However, the role of exosomes in cancer progression is largely unknown. Several studies have shown that exosomes are key components of the tumor microenvironment and regulate tumor growth and metastasis (Kalluri, 2016; Melo et al., 2014). Exosomes derived from tumors have been shown to promote tumor growth and cancer progression (Wu et al., 2023; Becker et al., 2016).

In this study, we examined the relationship between SARS-CoV-2 and hepatocellular carcinoma. The results showed that SARS-2-S promoted the development of hepatocellular carcinoma by inducing the secretion of exosomes after syncytium formation. This study provides new targets and treatment ideas for cancer patients with COVID-19.

## Materials and methods

### Reagents

Fetal bovine plasma (A3160901) was purchased from Gibco (New York, USA). Dulbecco’s modified Eagle’s medium (DMEM, high glucose, 11965092) was purchased from Thermo Fisher Scientific (Waltham, MA, USA). The protease inhibitor cOmplete™ Cocktail (4693116001) was purchased from Roche (Basel, Switzerland). β-galactosidase Reporter Gene Test Kit (RG0036) and Cell Counting Kit-8 (C0038) were purchased from Beyotime (Shanghai, China). Crystal violet (C8470) was purchased from Solarbio (Beijing, China). A Hieff^®^ Quick Exosome Isolation Kit (for Cell Culture Media) (41201ES50) was purchased from Yeasen (Shanghai, China).

### Antibodies

An anti-β-tubulin antibody (AC030, 1:2000 dilution) was purchased from ABclonal (Wuhan, China). Anti-ACE2 (21115-1-AP, 1:500 dilution), anti-SARS-CoV-2 S protein (28867-1-AP, 1:1000 dilution), anti-TSG101 (28283-1-AP, 1:2000 dilution), anti-CD9 (20597-1-AP, 1:1000 dilution) and anti-JAK1 (66466-1-Ig, 1:1000 dilution) were purchased from Proteintech (Wuhan, China). Anti-Na^+^/K^+^-ATPase (ab76020, 1:1000 dilution) and anti-p-STAT3 (ab76315, 1:2000 dilution) were purchased from Abcam (Illinois, USA). Anti-CD63 (bsm-52384R, 1:500 dilution) was purchased from Bioss (Beijing, China). Anti-STAT3 (sc-7179, 1:200 dilution) was purchased from Santa Cruz Biotechnology (Texas, USA).

### PDX mouse model

Female immunodeficient NOD/SCID mice (6 to 8 weeks old) (GemPharmatech, Nanjing, China) were used to establish the PDX mouse model at Crown Bioscience (Zhongshan, China). Fresh liver tumor tissues (Asian, 70 years old, male, moderately to poorly differentiated adenocarcinoma)were cut into small pieces (2-3 mm in diameter) and then subcutaneously transplanted into the right flanks of the mice. Lentiviruses encoding the vesicular stomatitis virus G glycoprotein (VSV-G) or the SARS-CoV-2 spike protein (SARS-2-S) were manufactured by VectorBuilder (Guangzhou, China). The pMD2.G, psPAX2 and pLV-eGFP-VSV-G or pLV-eGFP-SARS-2-S vectors were transfected into HEK293T cells. After 48 h, the lentiviruses were harvested, concentrated at 50,000 ×g in 20% (w/v) sucrose solution and titrated. Once the tumors grew to a size of 160-190 mm^3^, the lentiviruses were injected intratumorally (i.t.) at the indicated times (1.2 × 10^7^ TU/mouse) twice per week for two weeks. Tumors were collected for further analysis when the mice were sacrificed at the end of the experiment. The animal studies were performed according to the SOP in a specific pathogen-free facility under the approval of the Institutional Animal Care and Use Committee.

### Immunohistochemistry

Tumor tissues were fixed with 4% paraformaldehyde solution for 24 h. Then, the tissues were subjected to gradient ethanol dehydration, paraffin embedding, slicing, antigen repair, inactivation of endogenous enzyme activity, blocking, and primary antibody incubation overnight at 4°C. The tissues were incubated with HRP-labeled antibodies at room temperature for 1 h. The tissues were incubated with 1× DAB for 5 min, and the reaction was immediately stopped when a brown color appeared. The tissues were placed on cover slides and observed by light microscopy.

### Immunofluorescence analysis

The tissue sections were permeabilized with 0.3% Triton X-100 in PBS and blocked with 5% (w/v) bovine serum albumin in PBS for 1 h at room temperature. Then, the sections were incubated with primary antibodies in PBS for 1 h and incubated with fluorophore-conjugated antibodies in PBS for 30 min. Finally, the sections were labeled with DAPI solution.

### Cell culture

A549, Huh-7, H22 and HEK293T cells were obtained from the American Type Culture Collection (ATCC, Rockville, MD, USA). All the cell lines were mycoplasma free and incubated in DMEM supplemented with 10% FBS, 100 U/mL penicillin, and 0.1 mg/mL streptomycin at 37°C in a humidified atmosphere with 5% CO_2_.

A549 cells were infected with lentiviruses encoding ACE2 or SARS-2-S and selected with puromycin (4 μg/mL) for two weeks.

### Cell–cell fusion assay and collection of cellular supernatants

A549 cells overexpressing ACE2 (A549-ACE2) or SARS-2-S (A549-S) were cultured to 60% confluence. Then, A549-ACE2 and A549-S cells were mixed in equal proportions. After 12 h, the medium was replaced with serum-free medium, and the cells were cultured for 36 h. The supernatants were then harvested.

### Quantification of syncytial formation *in vitro*

To quantify the level of syncytial formation *in vitro*, we used α-complementation of the lacZ system based on β-galactosidase (Theuerkauf et al., 2021). First, SARS-2-S and lacZ (△11-41 aa) were overexpressed in A549 cells to construct producer cells, and ACE2 and lacZ (1-56 aa) were overexpressed in A549 cells to construct target cells. Upon syncytial formation, complementation of lacZ (△11-41 aa) with lacZ (1-56 aa) inside the syncytium results in the formation of active β-galactosidase. The β-galactosidase activity was measured using the β-galactosidase Reporter Gene Test Kit according to the manufacturer’s instructions.

### Qualitative of syncytial formation *in vivo*

Immunofluorescence was used for qualitative of syncytial formation *in vivo*. In tissue sections, staining for the NA^+^/K^+^-ATP enzyme was indicated the cell membrane, and mouse hepatocytes with more than three nuclei were identified as syncytia.

### Exosome purification and verification

The A549-ACE2 and A549-S cell supernatants were mixed in equal proportions in the control group, and the syncytial supernatant was used as the experimental group. The collected supernatants were centrifuged at 3,000 × g for 10 min and transferred to new 50 ml centrifuge tubes to remove cell fragments. Exosomes were purified with a Hieff ^®^Quick Exosome Isolation Kit (for Cell Culture Media). The particle diameter and concentration of exosomes were measured by Nanoparticle tracking analysis (NTA). The total protein concentration of isolated exosomes was determined by the BCA protein quantification kit. After loading equal amounts of exosomes, exosomal markers CD9, CD63, and TSG101 were analyzed by Western blotting. The morphology of isolated exosomes was evaluated by scanning electron microscopy (SEM).

### Colony formation assay

Huh-7 cells were plated in 6-well plates with 2000 cells in each well, and 3 replicate wells were used for each group. After the cells had attached, a cell culture chamber with 0.4 μm pores was placed in each well. A549-ACE2, A549-S and A549-ACE2+A549-S cells were plated in the chambers in equal proportions. The cells in the chambers were cocultured with the cells in 6-well plates for 2 weeks. Then, the Huh-7 cells were fixed with 4% paraformaldehyde for 15 min and stained with 0.5% (w/v) crystal violet for 20 min. The number of colonies was counted after the images were acquired.

### Wound healing assay

Huh-7 cells were plated in 6-well plates. After the cells had attached, a cell culture chamber with 0.4 μm pores was placed in each well. A549-ACE2, A549-S and A549-ACE2+A549-S cells were plated in the chambers in equal proportions. When the Huh-7 cells were 90-95% confluent, the monolayer was evenly scratched with a sterile 200 μL pipette tip. The cells were washed twice with PBS, after which the medium was replaced with serum-free medium, and the cells were further cultured. Huh-7 cells were photographed under a low-power microscope at 0, 24 and 48 h. Wound healing was observed, and the migrating cells were analyzed by ImageJ software.

### Transwell migration assay

Huh-7 and H22 cells in the logarithmic growth phase were starved overnight. The cells were diluted to 5×10^5^/mL. The concentration of exosomes derived from control cells (Ctr-Exos) or syncytia (Syn-Exos) was measured by NTA and adjusted to 2×10^10^ particles/mL. Huh-7 or H22 cell suspensions (200 μL) were evenly mixed with 40 μL of Ctr-Exos or Syn-Exos. The mixed cell suspensions (240 μL) were added to the chambers. DMEM containing 20% FBS (600 μL) was added to the 24-well plates. The cells were incubated at 37°C for 48 h. Then, the cells were fixed with 4% paraformaldehyde for 15 min and stained with 0.5% (w/v) crystal violet for 20 min. The chambers were gently wiped with cotton swabs to remove cells that had not migrated. The migrated cells were observed and photographed with a microscope and analyzed by ImageJ software.

### Cell proliferation assay

Huh-7 cells in the logarithmic growth phase were digested and suspended in complete medium. The cells were diluted to 1×10^5^/mL. Huh-7 cell suspensions (80 μL) were evenly mixed with 20 μL of Ctr-Exos or Syn-Exos. The mixed cell suspensions (100 μL) were added to 96-well plates. The cells were incubated at 37°C for 48 h. Then, CCK-8 solution (10 μL) was added to each well. After 4 h of incubation, the absorbance at 450 nm was measured by an enzyme labeling instrument.

### WB analysis

NP40 lysis buffer containing a protease inhibitor was added to the cells and tissues. The lysate was ultrasonicated in an ice bath. Total proteins were extracted from cells and tissues by centrifugation (12,000 r, 10 min, 4°C). After the addition of 5× SDS−PAGE protein sample buffer, the cell lysates were denatured at 100°C for 10 min. The total proteins were separated by SDS−PAGE. The proteins were transferred to PVDF membranes by the wet transfer method. The PVDF membranes were blocked with 5% (w/v) skim milk for 1 h. The membranes were incubated with primary antibodies at room temperature for 2 h and then with HRP-labeled antibodies at room temperature for 1 h. The lanes were visualized with a chemiluminescence image analyzer. β-Tubulin was used as a reference, the gray value of each band was analyzed by ImageJ software, and the relative expression of each protein was calculated.

### Database search and statistical analysis

Differentially expressed proteins (DEPs) were identified using a threshold fold change of ≥2 and a P value of <0.05. DEPs were analyzed by principal component analysis and PCA diagram was made. The volcanic plot is made with the fold change (log2 transformed) as the horizontal axis and the P value (-log10 transformed) as the longitudinal axis. DEPs were further subjected to GO enrichment analysis (http://geneontology.org/). The upregulated tumorigenic proteins and downregulated tumor suppressor proteins were shown by heatmaps. The original proteomics data has been uploaded to iProX (Project ID: IPX0008576000).

### Statistical analysis

GraphPad Prism 8.0 was used to plot the data and perform the statistical calculations. Differences between two independent samples were evaluated using two-tailed Student’s t tests. A P value < 0.05 was considered to indicate statistical significance. Significance values were set as follows: ns (not significant), P > 0.05; *, P < 0.05; **, P < 0.01; and ***, P < 0.001.

## Results

### Syncytia promote the proliferation and migration of hepatocellular carcinoma cells

**Figure 1:**
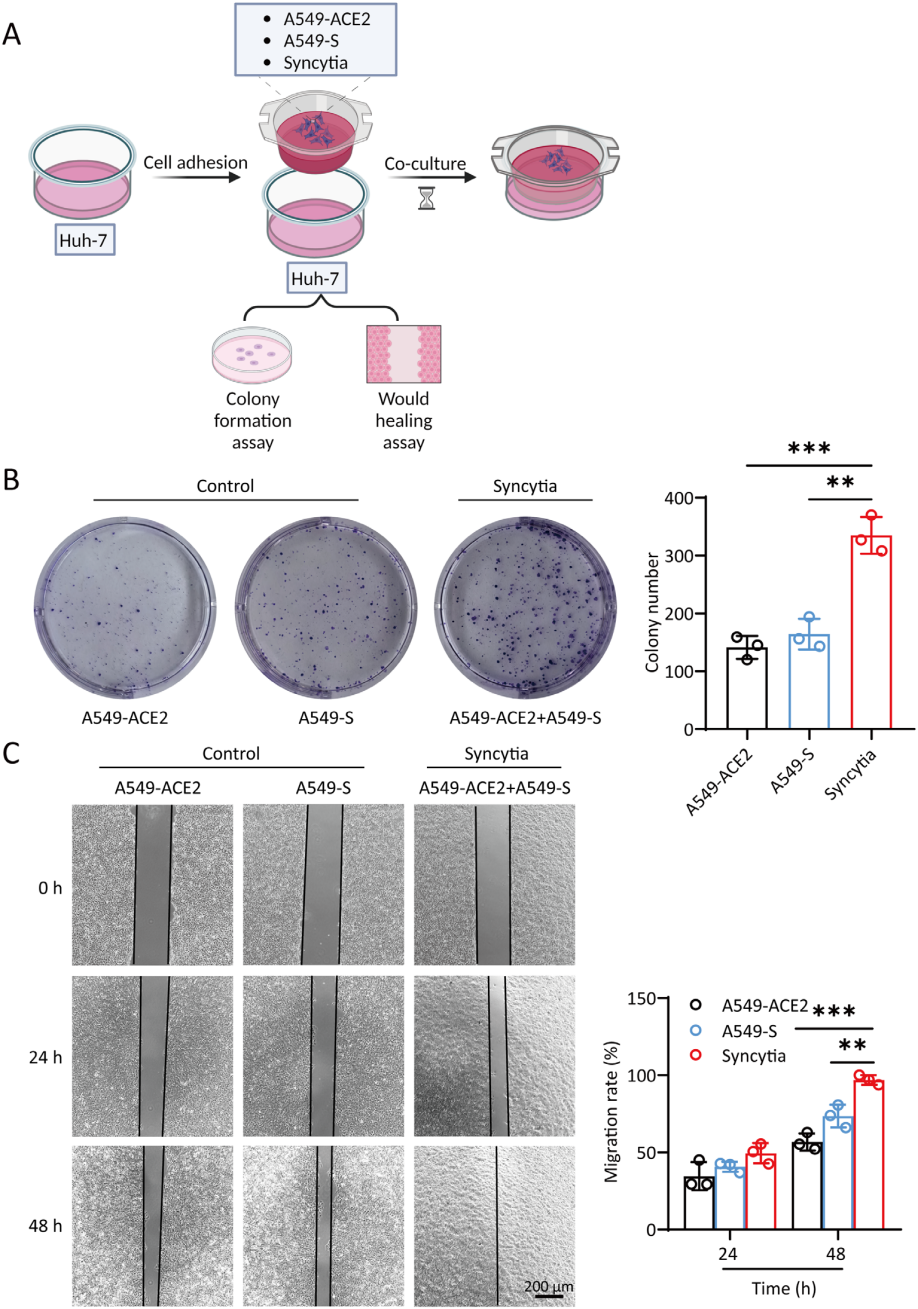
Syncytia promote the proliferation and migration of hepatocellular carcinoma cells. **(A)** Illustration of Huh-7 cells cocultured with A549-ACE2/A549-S/syncytia. (Created with BioRender.com). **(B)** Crystal violet staining of Huh-7 cells. For colony formation assays, Huh-7 cells were cocultured with A549-S or A549-ACE2 cells and syncytia for 2 weeks in 6-well plates. **(C)** Cell migration was measured by a wound healing assay. Huh-7 cells were cocultured with A549-S cells, A549-ACE2 cells and syncytia for 0 h, 24 h, or 48 (h) The relative migration area was analyzed by ImageJ. The data are presented as the mean ± SEM and were analyzed with two-sided Student’s t tests. **P < 0.01; ***P < 0.001.

First, we examined the effect of SARS-2-S on hepatocellular carcinoma cells. We hypothesized that the relationship between SARS-CoV-2 and tumors may be related to syncytium formation induced by SARS-2-S. We constructed A549 cell lines (A549-ACE2/A549-S) overexpressing the ACE2 and SARS-2-S proteins (**Supplementary Figure S1A**). A549-ACE2 (1-56 aa) and A549-S (△11-41 aa) cells were plated in the 6-well plate in equal proportions. After incubation for different times, the activity of β-galactosidase was measured. The results showed that the number of syncytia gradually increased as the culture time increased (**Supplementary Figure S1B**). These cell lines were used to study the effects of syncytia on the proliferation and migration of the hepatocellular carcinoma cell line Huh-7 (**Figure 1A**). Huh-7 cells were plated in 6-well plates at 2000 cells per well. A549-ACE2, A549-S or A549-ACE2+A549-S cells were added to the cell culture chamber and cocultured with Huh-7 cells, after which a colony formation assay was performed. The results showed that syncytia formed in a large area of A549-ACE2+A549-S cells in the cell culture chamber (**Supplementary Figure S1C**). Syncytia promoted the formation of more colonies of Huh-7 cells, and significantly more colonies formed than those cocultured with A549-ACE2 or A549-S cells (**Figure 1B**). Under these conditions, Huh-7 cells were cocultured with the A549 cell line. After 12 h, a wound healing assay was performed. Cell migration was observed at 0 h, 24 h and 48 h. The migration induced by syncytia was significantly greater than that induced by A549-ACE2 or A549-S cells (**Figure 1C**). These results suggest that the formation of syncytia promotes the proliferation and migration of hepatocellular carcinoma cells.

### Protein expression signatures in Syn-Exos

**Figure 2:**
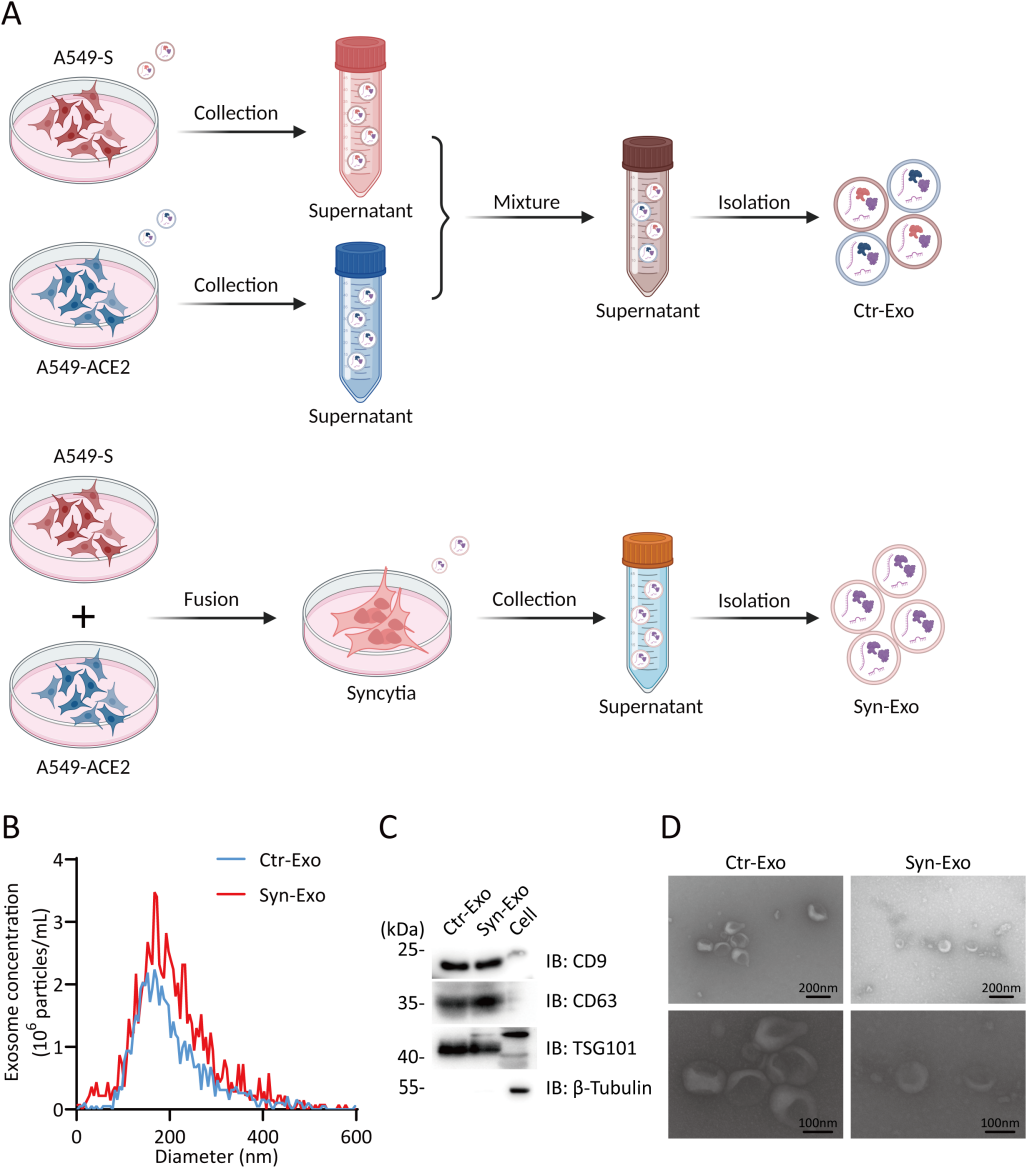
Analysis of exosome purity. **(A)** Illustration of the isolation of Ctr-Exos and Syn-Exos. (Created with BioRender.com). **(B)** Phenotypic analysis of Ctr-Exos and Syn-Exos using NTA. The X-axis represents the vesicle diameter, and the Y-axis represents the vesicle concentration (particles/ml). **(C)** WB analysis was performed to examine typical exosomal biomarkers (CD9, CD63 and TSG101) in Ctr-Exos and Syn-Exos. **(D)** Representative SEM images of Ctr-Exos and Syn-Exos.

**Figure 3:**
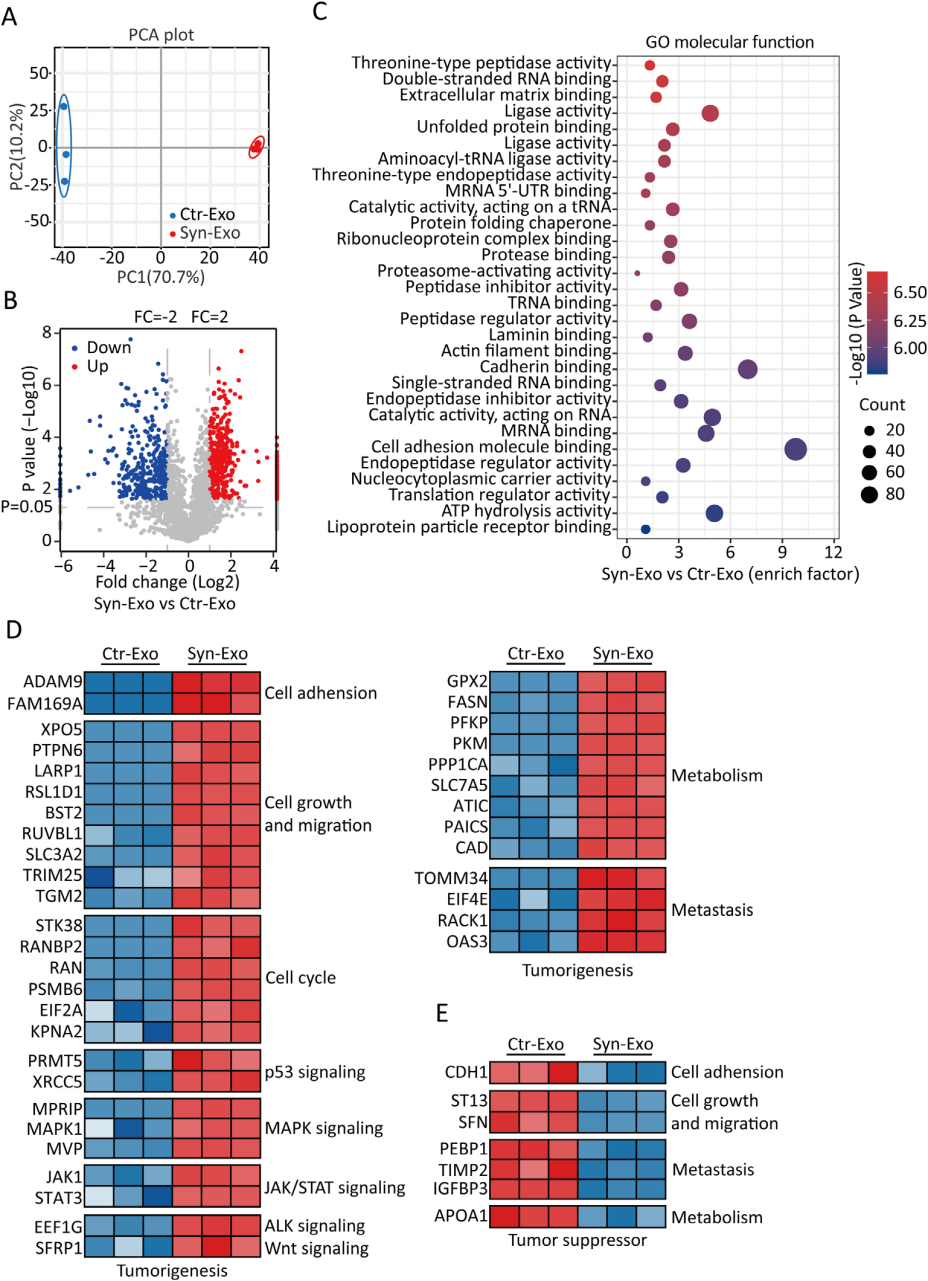
Protein expression signatures of Syn-Exos. **(A)** PCA results. PCA was based on the gene expression patterns of the Ctr-Exo (blue) and Syn-Exo (red) groups. In all plots, each point represents one sample. **(B)** Volcano plot showing Syn-Exos vs. Ctr-Exos. The red and blue plots represent proteins that were significantly upregulated and downregulated by twofold, respectively, in exosomes secreted by syncytia. A volcano plot was generated, with the log2 value of the fold change on the horizontal axis and the log10 value of the P value on the vertical axis. The data were analyzed with two-sided Student’s t tests. **(C)** Pathways enriched in the differentially expressed proteins in Syn-Exos according to GO term analysis of molecular function (https://www.Metascape.org). **(D)** Heatmap showing the upregulated tumorigenesis-related proteins in Syn-Exos. **(E)** Heatmap showing the downregulated tumor suppressor proteins in the Syn-Exos group.

A549-ACE2 and A549-S cells were cultured individually. The supernatants were mixed and used as a control. Exosomes from the supernatants of syncytia or control cells were extracted (**Figure 2A**). The purity of the exosomes was verified by NTA, WB analysis and SEM. NTA also revealed that the sizes of the exosomes in the two groups were similar (**Figure 2B**). WB analysis revealed that the exosomes in both groups expressed CD9, CD63 and TSG101. The exosomes had no cellular contamination (**Figure 2C**). SEM revealed that the exosomes in the two groups were structurally intact and morphologically similar (**Figure 2D**). Next, we inferred the possible mechanisms by which exosomes promote tumor proliferation and metastasis from the proteomic analysis of exosomes. Principal component analysis (PCA) revealed that the Syn-Exos were well separated from the Ctr-Exos (**Figure 3A**). Compared with those in Ctr-Exos, 829 differentially expressed proteins were found in Syn-Exos, 501 of which were significantly upregulated and 328 of which were significantly downregulated (**Figure 3B**). GO enrichment analysis revealed that the Syn-Exos significantly affected cell adhesion, cell adhesion molecule expression and other biological processes (**Figure 3C**). In particular, compared with those in Ctr-Exos, the levels of tumorigenic proteins in Syn-Exos, such as genes related to cell adhesion (ADAM9 and FAM169A), cell growth and migration (XPO5, PTPN6, LARP1, RSL1D1, BST2, RUVBL1, SLC3A2, TRIM25 and TGM2), the cell cycle (STK38, RANBP2, RAN, PSMB6, EIF2A and KPNA2), p53 signaling (PRMT5 and XRCC5), MAPK signaling (MPRIP, MAPK1 and MVP), JAK/STAT signaling (JAK1 and STAT3), ALK signaling (EEF1G), Wnt signaling (SFRP1), metabolism (GPX2, FASN, PFKP, PKM, PPP1CA, SLC7A5, ATIC, PAICS and CAD) and metastasis (TOMM34, EIF4E, RACK1 and OA53), were significantly increased (**Figure 3D**). Moreover, the levels of tumor suppressor proteins, such as those involved in cell adhesion (CDH1), cell proliferation and metastasis (ST13 and SFN), metastasis (PEBP1, TIMP2, and IGFBP3) and metabolism (APOA1), were significantly decreased in Syn-Exos (**Figure 3E**). These results suggest that Syn-Exos may promote tumor cell proliferation and metastasis.

### Syn-Exos promote the proliferation and migration of hepatocellular carcinoma cells

**Figure 4:**
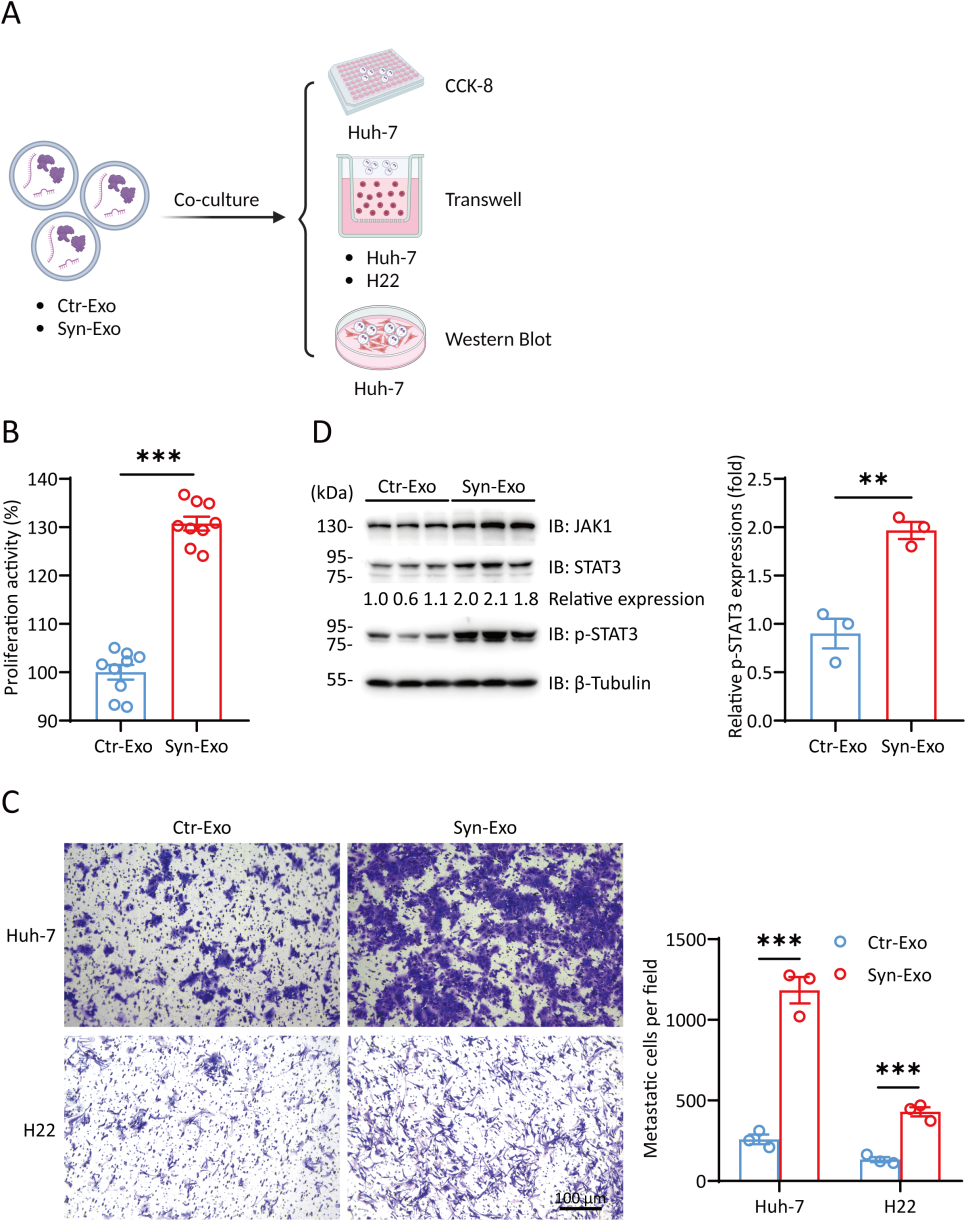
Syn-Exos promote the proliferation and migration of hepatocellular carcinoma cells. **(A)** Illustration of Huh-7/H22 cells cocultured with Ctr-Exos or Syn-Exos. (Created with BioRender.com). **(B)** A CCK-8 assay was performed to measure the proliferation of Huh-7 cells. Huh-7 cells were treated with Ctr-Exos or Syn-Exos for 24 (h) **(C)** Cell migration was measured by a transwell migration assay. Huh-7 cells or H22 cells were treated with Ctr-Exos or Syn-Exos for 48 h, respectively. The migrating cells were stained with crystal violet and observed by light microscopy. The number of migrating cells in each group was counted in three fields of view. **(D)** The expression of JAK1, STAT3 and p-STAT3 was measured by Western blotting. The data are presented as the means ± SEMs and were analyzed with two-sided Student’s t tests. **P < 0.01; ***P < 0.001.

We further verified the role of Syn-Exos in hepatocellular carcinoma cells by Cell Counting Kit-8 (CCK-8) assays, Transwell migration assays and WB analysis. (**Figure 4A**). First, we cocultured Ctr-Exos or Syn-Exos with Huh-7 cells in 96-well plates. The proliferation of Huh-7 cells was examined by CCK-8 assays. Syn-Exos significantly promoted the proliferation of Huh-7 cells (**Figure 4B**). Then, we cocultured Syn-Exos with Huh-7 or H22 cells in the cell chambers. A transwell migration assay was performed to examine the migration of tumor cells. Syn-Exos significantly promoted the migration of Huh-7 and H22 cells (**Figure 4C**). In the previous proteomics analysis, we screened a series of proteins that promoted the proliferation of tumor cells. Therefore, Ctr-Exos or Syn-Exos (200 μL) were cocultured with Huh-7 cells in 5 cm cell culture dishes for 48 h. The expression of tumorigenic proteins in Huh-7 cells was examined by WB analysis. Syn-Exos significantly increased the phosphorylation of STAT3 and activated the JAK1/STAT3 signaling pathway (**Figure 4D**). The mechanism by which Syn-Exos promote tumors may be mediated by the JAK1/STAT3 pathway.

### SARS-2-S promotes tumor growth in a PDX mouse model

**Figure 5:**
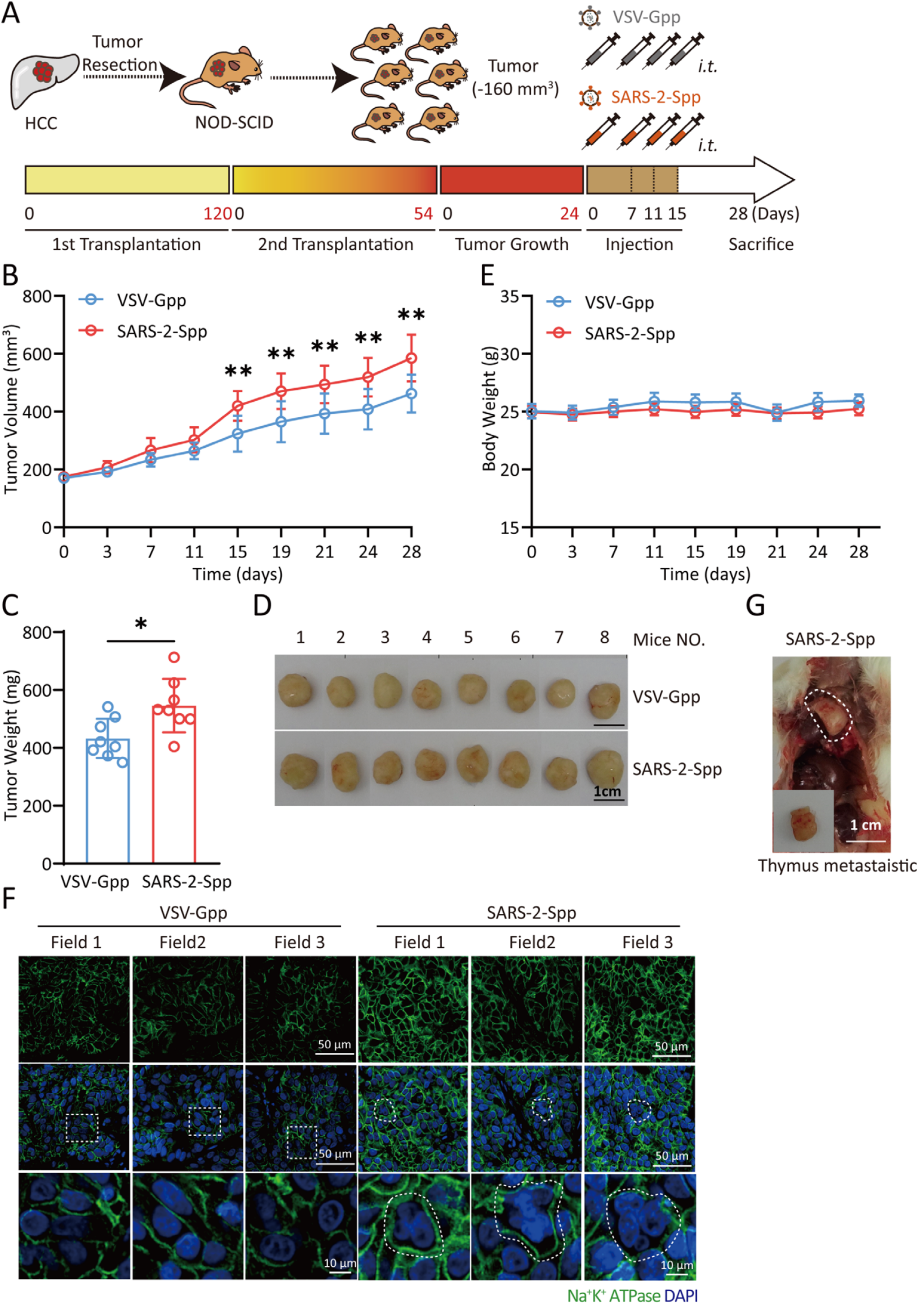
SARS-2-S promotes tumor growth in a PDX mouse model. **(A)** Illustration of the PDX mouse model. Cells obtained from resected carcinoma (HCC) tissue with high ACE2 staining were subcutaneously injected into the backs of the mice, which were then injected with VSV-Gpp or VSV-SARS-2-S twice per week. **(B, C)** PDX tumor volume **(B)** and terminal tumor weight **(C)**. **(D)** Images of tumors obtained from these mice. **(E)** PDX mouse body weights. **(F)** One thymus metastatic foci was observed in one SARS-2-S-treated mouse. **(G)** Immunofluorescence analysis of multinucleated cells in tumor tissue stained with anti-Na+/K+-ATPase in tumor sections from SARS-2-Spp-treated mice. The images are representative of three independent experiments. Statistical significance was determined with two-tailed Student’s t tests **(C)**, one-way ANOVA and Bonferroni *post hoc* analysis **(B)**. *P < 0.05; **P < 0.01.

Our results explain the function of Syn-Exos *in vitro*. Next, we examined their effects on tumors *in vivo*. We used PDX mice to evaluate the pathological role of SARS-2-S. We selected human hepatocellular carcinoma (HCC) tissue with high expression of ACE2 to construct a PDX mouse model (**Supplementary Figure S2A**). Pseudotyped lentiviruses bearing SARS-2-S (SARS-2-Spp) or VSV-Gpp were verified in A549 cells (**Supplementary Figure S2B**). Then, we intratumorally injected SARS-2-Spp or VSV-Gpp into PDX mice and examined the growth rate and size of the tumors. SARS-2-Spp or VSV-Gpp (1.2×10^7^ TU/mouse) was injected intratumorally twice per week for 2 weeks (**Figure 5A**). Compared with that in the VSV-Gpp group, the tumor volume in the SARS-2-Spp group was significantly greater (**Figure 5B**, 5D), the tumor weight was significantly greater (**Figure 5C**), and the tumor progressed more rapidly. However, there was no difference in body weight between the two groups (**Figure 5E**). The results showed that SARS-2-S promoted tumor growth in PDX mice. Then, paraffin sections of tumor tissues obtained from the PDX mice were examined by immunofluorescence staining. Staining for the NA^+^/K^+^-ATP enzyme (cell membrane) and with DAPI showed that there were more syncytia in the tumors in the SARS-2-Spp group, and there were fewer syncytia (**Figure 5F**) in the tumors in the VSV-Gpp group. These results showed that the increase in syncytia was closely related to tumor tissue enlargement, which was consistent with the findings of previous cell experiments. Notably, a thymic metastatic lesion (**Figure 5G**) was found on a mouse treated with SARS-2-Spp. In conclusion, we believe that SARS-2-S can promote tumor growth in PDX mice.

## Discussion

COVID-19 has become a sudden public health problem worldwide. The number of people infected with SARS-CoV-2 and the extent to which the virus spread far exceeded those of other epidemic diseases in recent years. Certainly, in some groups, such as cancer patients infected with COVID-19, disease severity and mortality are significantly increased (Saini et al., 2020; Rivera et al., 2020; Zhang et al., 2021). The increase in mortality in these individuals may be closely related to the increase in the SARS-CoV-2 viral load (Westblade et al., 2020). In this study, we demonstrated the unique mechanism by which SARS-2-S promoted the progression of hepatocellular carcinoma. Several findings support this conclusion. First, the formation of syncytia promoted the proliferation and migration of Huh-7 cells. Second, the expression of tumorigenic proteins was significantly upregulated, and the expression of tumor suppressor proteins was significantly downregulated in the Syn-Exos group. Third, Syn-Exos activated the JAK1/STAT3 signaling pathway and promoted the proliferation and migration of Huh-7 and H22 cells. Finally, the tumor proliferation rate was significantly increased in PDX mice injected with SARS-2-Spp. The number of syncytia induced by SARS-2-S was increased in tumor tissues. In summary, SARS-2-S plays a significant role in tumors. Low-titer viruses such as SARS-CoV-2 use syncytia to escape the host’s immune defenses. These viruses continue to replicate and infect other cells to form additional syncytia. These syncytia continuously secrete exosomes, which are crucial in the tumor microenvironment. Cancer patients who do not develop inflammatory cytokine storms or ARDS in response to SARS-CoV-2 infection may still face a risk of rapid tumor proliferation and metastasis due to COVID-19.

COVID-19 has attracted increasing attention due to potential long-term sequelae outside the lungs (Gupta et al., 2020). Liver injury is one of the main extrapulmonary manifestations. Approximately one-fifth of COVID-19 patients have abnormalities in liver function (Mao et al., 2020; Gracia-Ramos et al., 2021). In cases of liver injury, the expression of ACE2 is increased in hepatocytes (Herath et al., 2007). In addition, high levels of SARS-CoV-2 particles are observed in the cytoplasm of liver cells in COVID-19 patients. Most viral particles have an intact envelope. These findings show that SARS-CoV-2 can not only enter liver cells but also replicate in liver cells. Distinct binuclear or multinucleated syncytial hepatocytes have also been identified in liver tissue (Wang et al., 2020). Therefore, this study used hepatocellular carcinoma as an example to examine the development of liver cancer in patients with COVID-19. Unfortunately, this study has certain limitations. First, the composition of exosomes is complex and may involve multiple factors, such as proteins, lipids, and nucleic acids (Kalluri and LeBleu, 2020). It is still unclear which components within exosomes activate the JAK1-STAT3 pathway to promote tumors. Second, whether Syn-Exos activate other tumor-promoting pathways is worth further exploration.

In conclusion, we provide evidence that Syn-Exos induced by SARS-2-S can promote the proliferation and metastasis of hepatocellular carcinoma cells. Our findings reveal a new mechanism for the development of liver cancer in patients with COVID-19. In future studies, Syn-Exos could be used as targets for COVID-19 research. These findings could lead to treatments for cancer patients suffering from COVID-19. There are also broad prospects for developing related antitumor drugs based on this target.

## Data Availability

The datasets presented in this study can be found in online repositories. The names of the repository/repositories and accession number(s) can be found in the article/**Supplementary Material**.

## Supplementary material

Supplementary Figure 1 Verification of the A549 cell line and syncytia. **(A)** A549 cell lines overexpressing SARS-2-S and ACE2 were verified by Western blotting. **(B)** Quantification of syncytial formation *in vitro*. **(C)** The morphology of A549-ACE2, A549-S and syncytia was observed by light microscopy.

Supplementary Figure 2 The verification of tumor tissues and lentiviruses. **(A)** Immunohistochemical analysis of resected HCC tissues with ACE2 antibodies. **(B)** A549 cells were treated with SARS-2-Spp or VSV-Gpp for 24 h. The cells were collected and analyzed by Western blotting with an anti-S antibody. β-Tubulin was used as a loading control.
