# Supplementary figures and images for: Exosomes derived from syncytia induced by SARS-2-S promote the proliferation and metastasis of hepatocellular carcinoma cells
Source: Front Cell Infect Microbiol. 2025 Jan 8;14:1415356. doi: 10.3389/fcimb.2024.1415356 (PMC11750861; doi:10.3389/fcimb.2024.1415356)

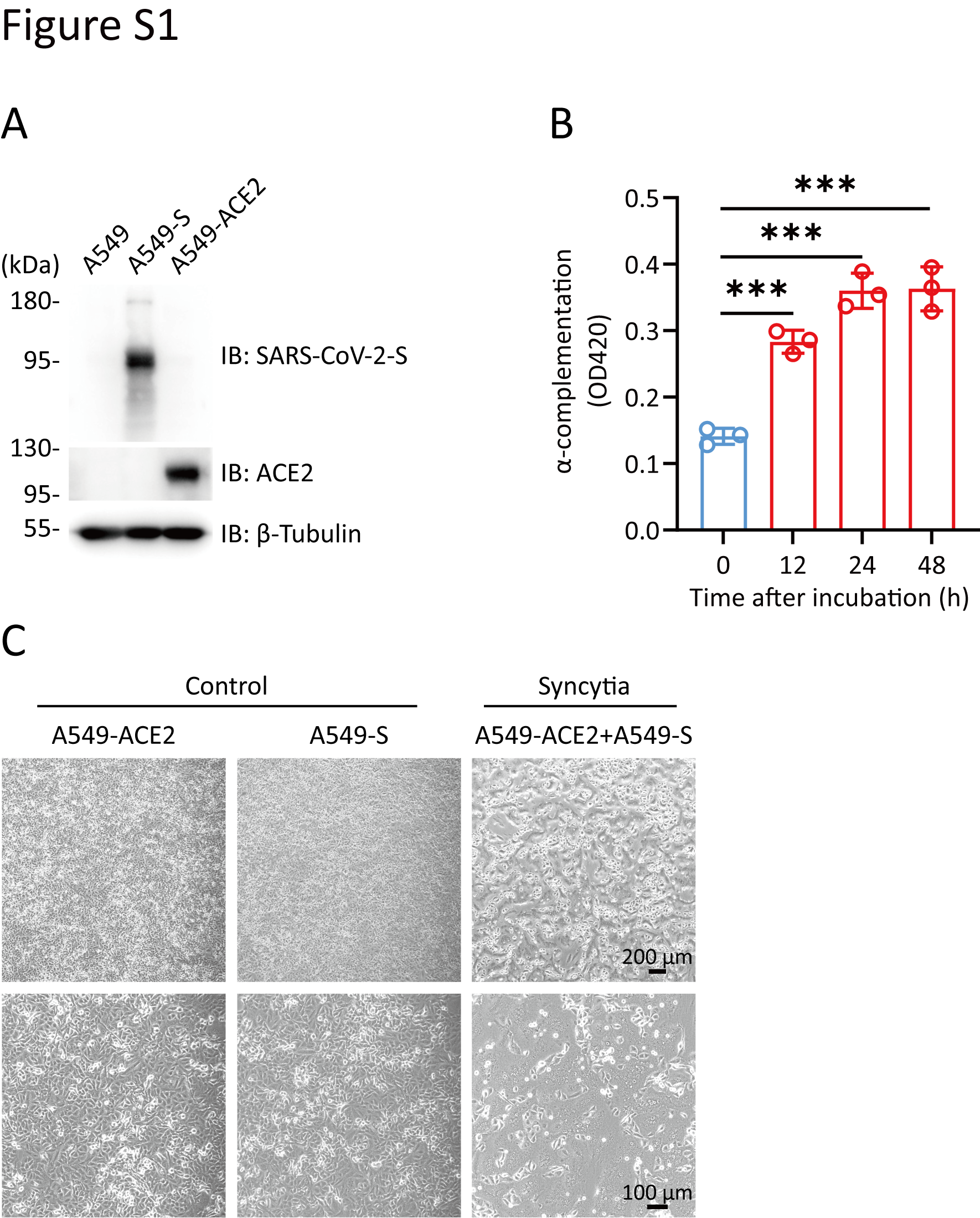

Supplement: Supplementary Figure 1 — Verification of the A549 cell line and syncytia. (A) A549 cell lines overexpressing SARS-2-S and ACE2 were verified by Western blotting. (B) Quantification of syncytial formation in vitro. (C) The morphology of A549-ACE2, A549-S and syncytia was observed by light microscopy. [file Image1.tif]

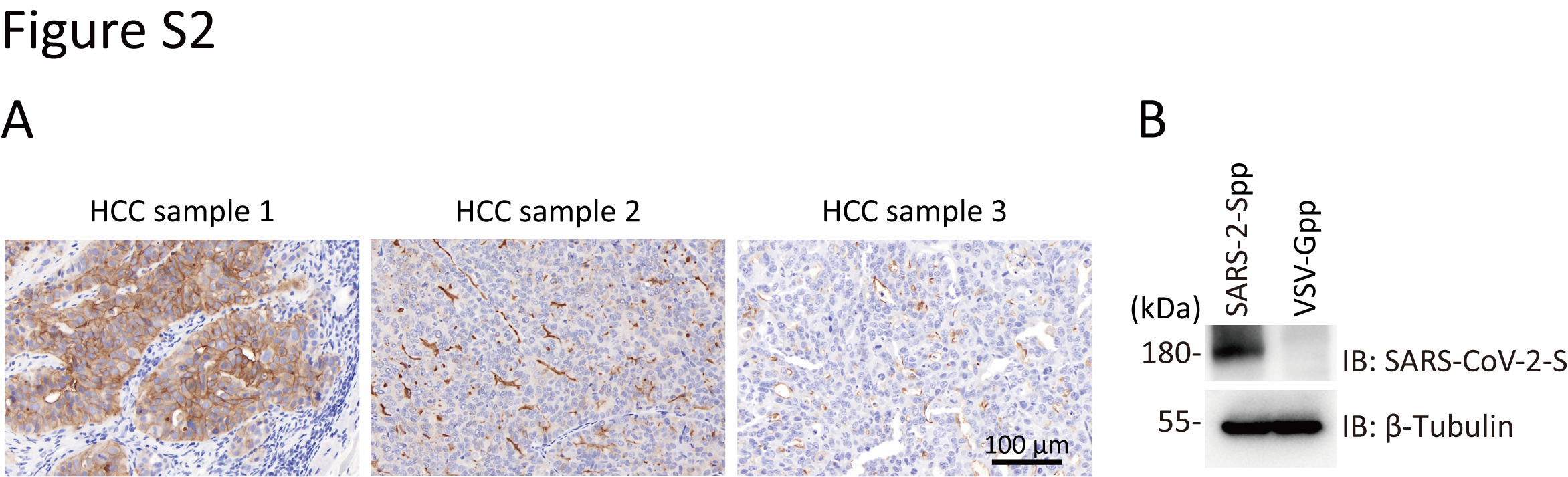

Supplement: Supplementary Figure 2 — The verification of tumor tissues and lentiviruses. (A) Immunohistochemical analysis of resected HCC tissues with ACE2 antibodies. (B) A549 cells were treated with SARS-2-Spp or VSV-Gpp for 24 h. The cells were collected and analyzed by Western blotting with an anti-S antibody. β-Tubulin was used as a loading control. [file Image2.tif]
